# Supplementary material for: FoxP3+ Regulatory T-Cell Quantities in Nodal T-Follicular Helper Cell Lymphomas and Peripheral T-Cell Lymphomas Not Otherwise Specified and Their Impact on Overall Survival
Source: Cancers (Basel). 2024 Nov 29;16(23):4011. doi: 10.3390/cancers16234011 (PMC11640135; doi:10.3390/cancers16234011)
Supplement: Supplementary file 1 [file cancers-16-04011-s001.zip › cancers-3258119-supplementary.pdf]

Table S1. Panel of antibodies for IHC staining.

Primary Ab	Provider	Clone	Ag retrieval	Ab dilution	Ab incubation (min)	Visualization (detection kit)
Bcl-6	Cell Marque	GI191E/A8	CC1	1:800	60	OptiView
CD2	Epitomics	EP222	CC1	1:100	60	OptiView
CD3	Dako	polyclonal	CC1	1:400	60	OptiView
CD4	Cell Marque	SP35	CC1	1:10	32	OptiView
CD5	Novocastra	4C7	CC1	1:400	60	OptiView
CD7	Cell Marque	MRQ-56	CC1	1:200	60	OptiView
CD8	Dako	C8/144B	CC1	1:100	60	OptiView
CD10	Novocastra	56C6	CC1	1:20	60	OptiView
CD20	Dako	L26	CC1	1:500	60	OptiView
CD21	Cell Marque	EP3093	CC1	1:200	60	OptiView
CD23	Cell Marque	1B12	CC1	1:50	32	OptiView
CD30	Cell Marque	Ber-H2	CC1	1:50	60	OptiView
CD56	Cell Marque	MRQ-42	CC1	1:200	60	OptiView
CD278 (ICOS)	Abcam	SP98	CC1	1:50	60	OptiView
CXCL13	RD systems	53602	P1	1:50	60	OptiView
GATA3	Cell Marque	L50-823	CC1	1:400	60	OptiView
GranB	Dako	GrB-7	CC1	1:10	60	OptiView
Ki-67	Dako	MIB-1	CC1	1:200	60	OptiView
p53	Dako	D0-7	CC1	1:3200	60	OptiView
PD-1	Cell Marque	MRQ-22	CC1	1:800	60	OptiView
Perforin	Cell Marque	MRQ-23	CC1	1:50	60	OptiView
TCRβ	Invitrogen	8A3	P1	1:100	60	OptiView
TCRδ	Santa Cruz Biot.	H-41	CC1	1:50	60	OptiView
TIA-1	Beckman Coulter	2G9A10F5	CC1	1:2000	60	OptiView

Ab: antibody; Ag: antigen; CC1: cell conditioning solution.

Table S2. Results of a of Cox regression model (multivariate analysis) showing that the non-linear effect is not statistically significant (p=0.44).

Variables included	Chi-Square	d.f.	p-value
Treg value	1.54	2	0.46
Nonlinear effect	0.61	1	0.44
IPI risk groups	26.34	3	0.00
Diagnosis	0.54	4	0.97
TOTAL	42.14	9	0.00

IPI: international prognostic index.
